# Supplementary material for: HIV testing behaviors and willingness to receive oral rapid HIV testing among dental patients in Xi’an, China
Source: PLoS One. 2021 Mar 25;16(3):e0248585. doi: 10.1371/journal.pone.0248585 (PMC7993620; doi:10.1371/journal.pone.0248585)
Supplement: S1 Questionnaire — (DOCX) [file pone.0248585.s002.docx]

人群健康知识问卷

您好，我们是西安交通大学医学部的学生。为了解您对HIV快速检测的接受度，我们开展了本次问卷调查。这是不记名问卷，不会对您造成任何不良影响，仅需要占用您5-10分钟时间。

感谢您能参加我们的调查！

1. 您是哪一年出生的：________

2. 性别： ①男 ②女

3. 民族： ①汉族 ②回族 ③维吾尔族 ④其他

4. 文化程度：①小学及以下 ②初中 ③高中或中专 ④大专 ⑤本科 ⑥研究生及以上

5. 婚姻状况：①未婚 ②在婚 ③同居 ④离异或丧偶

6. 月收入（元）： ①1000以下 ②1000-2999 ③3000-4999

④5000-6999 ⑤7000-9999 ⑥10000及以上

7. 职业：①商业服务业员工、工人、农民 ②事业单位人员、公务员 ③医务人员

④教师、学生 ⑤个体商业户、私营企业主 ⑥军人 ⑦离退休 ⑧其他

8. 就诊科室：①牙体牙髓科 ②修复科 ③牙周粘膜科 ④正畸科 ⑤牙槽外科

⑥儿童牙病科 ⑦种植科 ⑧颌面外科 ⑨预防科 ⑩综合科 ⑪其他

9. 艾滋病感染者从外表能看出来吗？ ①不知道 ②能 ③不能

10. 与艾滋病感染者一起吃饭能传染吗？ ①不知道 ②能 ③不能

11. 与艾滋病感染者握手能传染吗？ ①不知道 ②能 ③不能

12. 与艾滋病感染者共用剃须刀能传染吗？ ①不知道 ②能 ③不能

13. 与艾滋病感染者共用牙刷能传染吗？ ①不知道 ②能 ③不能

14. 艾滋病可通过咳嗽、打喷嚏传染吗？ ①不知道 ②能 ③不能

15. 感染艾滋病的孕妇能把艾滋病传染给胎儿吗？ ①不知道 ②能 ③不能

16. 感染艾滋病的母亲能通过乳汁把病原体传染给婴儿吗？ ①不知道 ②能 ③不能

17. 艾滋病能通过被褥、毛巾等日常用品传染吗？ ①不知道 ②能 ③不能

18. 输入艾滋病感染者的血或血制品能感染艾滋病吗？ ①不知道 ②能 ③不能

19. 艾滋病能通过蚊虫叮咬传染吗？ ①不知道 ②能 ③不能

20. 与艾滋病感染者共用注射器吸毒能传染艾滋病吗？ ①不知道 ②能 ③不能

21. 与艾滋病感染者发生性行为能传染吗？ ①不知道 ②能 ③不能

22. 艾滋病有疫苗吗? ①不知道 ②有 ③没有

23. 艾滋病能治好吗？ ①不知道 ②能 ③不能

24. 您觉得自己有可能感染艾滋病吗？ ①有可能 ②没可能 ③不知道

25. 您做过艾滋病检测吗？ ①做过 ②没做过（跳至31题） ③不知道

26. 检测的地点是：①医院 ②疾控中心 ③血站/献血车 ④自己检测 ⑤其他

27. 检测的方法是：①采静脉血 ②采指尖血 ③采唾液（口腔黏膜渗出液） ④不知道

28. 检测的价格是：①免费 ②1-50元 ③51-100元 ④100元以上 ⑤不知道

29. 等待结果的时间是：①1小时以内 ②1-24小时 ③24小时以上 ④不知道

30. 您知道检测的结果吗？ ①知道 ②不知道

31. 采静脉血能检测艾滋病吗？ ①不知道（跳至33题） ②能 ③不能（跳至33题）

32. 等待结果的时间是：①不知道 ②1小时以内 ③1-24小时 ④24小时以上

33. 采指尖血能检测艾滋病吗？

①不知道（跳至35题） ②能 ③不能（跳至35题）

34. 等待结果的时间是： ①不知道 ②1小时以内 ③1-24小时 ④24小时以上

35. 用棉签在口腔黏膜上摩擦取渗出液能检测艾滋病吗？

①不知道（跳至37题） ②能 ③不能（跳至37题）

36. 等待结果的时间是：①不知道 ②1小时以内 ③1-24小时 ④24小时以上

37. 口腔黏膜快速检测的优点有: ①准确度高 ②出结果快 ③不用抽血

38. 如果口腔医生可以开展口腔黏膜渗出液快速检测，您愿意在治疗口腔疾病前检测吗？①愿意 ②不愿意(跳至40题) ③不确定(跳至41题)

39. 您愿意的原因是（可多选）：

①关注自身健康 ②避免传播他人 ③早发现早诊断早治疗 ④不用抽血

⑤操作简单方便 ⑥出结果快 ⑦其他__________

40. 您能接受的最高价格是：①免费 ②10元 ③30元 ④50元 ⑤80元及以上

41. 您不愿意的原因是（可多选）：

①认为自己健康不可能感染 ②耽误时间 ③担心结果不准确 ④害怕被人怀疑

⑤查出问题心理承受不了 ⑥跟自己的病没有关系 ⑦额外花钱

⑧其他________

42. 您不确定的原因是（可多选）：

①关注自身健康 ②避免传播他人 ③早发现早诊断早治疗 ④认为自己健康不可能感染

⑤耽误时间 ⑥担心结果不准确 ⑦害怕被人怀疑 ⑧查出问题心理承受不了

⑨跟自己的病没有关系 ⑩其他__________

*感谢您的配合，祝您生活愉快！*
