# Supplementary material for: HIV testing behaviors and willingness to receive oral rapid HIV testing among dental patients in Xi’an, China
Source: PLoS One. 2021 Mar 25;16(3):e0248585. doi: 10.1371/journal.pone.0248585 (PMC7993620; doi:10.1371/journal.pone.0248585)
Supplement: S2 Questionnaire — (DOCX) [file pone.0248585.s003.docx]

Questionnaire about HIV testing behavior and oral rapid HIV testing acceptance

Hello, we are students of the Medical Department of Xi'an Jiaotong University. In order to understand your HIV testing behavior and acceptance of oral rapid HIV testing, we carried out this survey. This questionnaire is anonymous and will not cause any adverse effects to you, that you only need to take 5-10 minutes. Would you like to join us?

Thank you for participating in our survey!

1. When were you born: _______

2. Gender: ①Male ②Female

3. Nationality: ①Han ②Hui ③Uyghur ④Others

4. Education level:

①Elementary school and below ②Junior high school ③High school or technical secondary school ④College ⑤Undergraduate ⑥Graduate and above

5. Marital status: ①Unmarried ②Married ③Cohabitation ④Divorced or widowed

6. Monthly income (yuan):

①Below 1000 ②1000-2999 ③3000-4999

④5000-6999 ⑤7000-9999 ⑥10000 and above
7. Occupation:

①Business service employees, workers, farmers ②Institution staff, civil servants ③Medical staff ④Teachers, students ⑤Individual business owners, private business owners ⑥Military ⑦Retired ⑧Others

8. Clinics: ①Endodontic ②Prosthodontics ③Periodontics ④Orthodontics

⑤Alveolus surgery ⑥Pediatric Dentistry ⑦Dental Implant

⑧Maxillofacial Surgery ⑨preventive dentistry ⑩Others

9. Do people living with AIDS/HIV show it from the outside?

①Don’t know ②Yes ③No

10. Can having a meal together with people living with AIDS/HIV infect?

①Don’t know ②Yes ③No

11. Is it contagious to shake hands with people living with AIDS/HIV?

①Don’t know ②Yes ③No

12. Can sharing a razor with people living with AIDS/HIV be contagious?

①Don’t know ②Yes ③No

13. Can sharing a toothbrush with people living with AIDS/HIV be contagious?

①Don’t know ②Yes ③No

14. Can AIDS/HIV be transmitted through coughing and sneezing?

①Don’t know ②Yes ③No

15. Can pregnant women infected with AIDS/HIV transmit HIV to their fetus?

①Don’t know ②Yes ③No

16. Can an AIDS-infected mother pass the pathogen to her baby through her milk?

①Don’t know ②Yes ③No

17. Can AIDS be transmitted through daily necessities such as bedding and towels?

①Don’t know ②Yes ③No

18. Can blood or blood products imported from HIV-infected people be infected with AIDS?①Don’t know ②Yes ③No

19. Can AIDS be transmitted through mosquito bites?

①Don’ t know ②Yes ③No

20. Can sharing syringes with people living with AIDS be able to transmit AIDS?

①Don’ t know ②Yes ③No

21. Is it contagious to have sex with an AIDS-infected person?

①Don’ t know ②Yes ③No

22. Is there a vaccine for AIDS?

①Don’ t know ②Yes ③No

23. Can AIDS be cured?

①Don’ t know ②Yes ③No

24. Do you think you may be infected with AIDS?

①It is possible ②Not possible ③Don't know

25. Have you been tested for AIDS?

①Done ②No (skip to question 31) ③Don’t know

26. The locations of testing were:

①Hospital ②CDC ③Blood station/blood donation vehicle ④Self-test ⑤Other

27. The testing methods are:

①Collect venous blood ②Collect blood from fingertips

③Collect saliva (oral mucosa exudate) ④Don’t know

28. The price of the test was:

①Free ②1-50 yuan ③51-100 yuan ④100 yuan or more ⑤Don’t know

29. The waiting time for the result was:

①Within 1 hour ②1-24 hours ③24 hours or more ④Don’t know

30. Do you know the results of the test? ① know ② don't know

31. Can venous blood be tested for AIDS?

①Don’t know (skip to question 33) ②Yes ③can’t (skip to question 33)

32. The waiting time for the result was (venous blood test):

①Don't know ②Within 1 hour ③1-24 hours ④24 hours or more

33. Can fingertip blood test be used to detect AIDS?

①Don’t know (skip to question 35) ②Yes ③can’t (skip to question 35)

34. The waiting time for the result was (fingertip blood test):

①Don't know ②Within 1 hour ③1-24 hours ④24 hours or more

35. Can oral mucosa exudate be tested for HIV?

①Don’t know (skip to question 37) ②Yes ③can’t (skip to question 37)

36. The waiting time for the result is:

①Don't know ②Within 1 hour ③1-24 hours ④More than 24 hours

(After a short introduction about Oral Rapid HIV Testing)

37. The advantages of Oral Rapid HIV Testing is:

①High accuracy ②Quick results ③No need to draw blood

38. If a dentist can carry out Oral Rapid HIV Testing, would you be willing to test it before treating oral diseases?

①Yes ②Unwilling (skip to 40 questions) ③Unsure (skip to 41 questions)

39. The reason you are willing is (multiple choices):

①Focus on one's own health ②Avoid spreading to others

③Early detection, early diagnosis and early treatment ④No need to draw blood

⑤Simple and convenient operation ⑥Quick results ⑦Others

40. The highest price you can accept for Oral Rapid HIV Testing is:

①free ②10 yuan ③30 yuan ④50 yuan ⑤80 yuan and above

41. The reason you are unwilling is (multiple choices are available):

①healthy enough ②Waste of time

③Worried about the inaccuracy of the test ④Worry about the discrimination

⑤Not able to bear the result (if reactive) ⑥Not related to their dental diseases

⑦Extra money ⑧Others________

42. The reason you are not sure is (multiple choices are available):

①healthy enough ②Waste of time

③Worried about the inaccuracy of the test ④Worry about the discrimination

⑤Not able to bear the result (if reactive) ⑥Not related to their dental diseases

⑦Extra money ⑧Others________

*THANKS FOR YOUR TIME AND WISH YOU A NICE DAY!*
